# Supplementary material for: Deletion of wfs1 Impairs Oligodendrocyte Precursor Cells Dorsal Distribution and Myelination Through the wfs1-hmgcs1 Axis in Zebrafish
Source: Biology (Basel). 2026 Aug 16;15(16):1402. doi: 10.3390/biology15161402 (PMC13510151; doi:10.3390/biology15161402)
Supplement: Supplementary file 1 [file biology-15-01402-s001.zip › File S1-Transcriptome sequencing data and differentially expressed genes (DEGs) in wfs1-- zebrafish at 5 dpf/2.KEGG/wfs1KOvsWT/UP/src/dre04510.html]

dre04510


- dre:564266

- Up regulated genes

564266(2.42311476837666)

- dre:570868

- Up regulated genes

570868\_1(5.31304404972279)

- dre:562529

- Up regulated genes

562529\_1(8.14060403944833)
- dre:563023

- Up regulated genes

563023\_1(8.87487844876329)

- dre:793158

- Up regulated genes

793158\_1(1.86160461223548)
- dre:556226

- Up regulated genes

556226\_1(6.36622858283894)

- dre:677750

- Up regulated genes

677750(1.37913343503877)
- dre:30429

- Up regulated genes

30429(3.98975094921512)

- dre:321070

- Up regulated genes

101882911(3.50047007681359)

- dre:100000252

- Up regulated genes

100000252\_3(1.94434154854557) 100000252(1.09163460881879)

- dre:564565

- Up regulated genes

564565(1.26204775967321)

- dre:100006675

- Up regulated genes

100006675(1.63301233543285)

- dre:436718

- Up regulated genes

436718(1.34229427553561)

- dre:321243

- Up regulated genes

321243\_1(9.00901912775666)
- dre:30037

- Up regulated genes

30037(1.55750167372461)
- dre:100005469

- Up regulated genes

100005469\_1(3.66353583031119)

- dre:245702

- Up regulated genes

245702(1.27755220735994)

- dre:796490

- Up regulated genes

796490\_2(1.58582846768856)
- dre:103909601

- Up regulated genes

103909601(4.16535952448207)

- dre:57934

- Up regulated genes

57934\_1(9.91304974129429)

Close
